# Companion animal adoption and relinquishment during the COVID-19 pandemic: The experiences of animal rescue staff and volunteers

**DOI:** 10.1017/awf.2024.15

**Published:** 2024-03-04

**Authors:** Grace A Carroll, Catherine Reeve, Alice Torjussen

**Affiliations:** 1 Animal Behaviour Centre, School of Psychology, Queens University Belfast, UK; 2 School of Engineering and Informatics, Brighton, University of Sussex, UK

**Keywords:** adoption, animal shelter, animal welfare, COVID-19, qualitative, relinquishment

## Abstract

There has been a paucity of research into the experiences of animal rescue staff and volunteers during COVID-19. The aim of this qualitative research was to explore the impact of the COVID-19 pandemic on companion animal rescue organisations and their staff and volunteers, and to develop a set of recommendations on how to reduce the risk to companion animal welfare during a crisis. Descriptive thematic analysis was used to explore the experiences of staff and volunteers from 28 animal rescue organisations, most of which were based in the UK. Other surveyed countries included Germany, the Republic of Ireland, France, Spain, the USA and Australia. We identify three key themes that reflect the challenges faced by pet rescue organisations during the pandemic: (1) impact on animals; (2) impact on identity; and (3) impact on organisational processes. Key recommendations include the promotion of co-operation and modifications within the sector, the need to understand, and change, detrimental pet-owner behaviours, and the need to clarify the identity of animal rescue organisations within the Government. Both positive and negative outcomes were experienced by animal rescue organisations during the COVID-19 pandemic. These findings should be considered for future crises and indeed the everyday operating procedures of companion animal rescue organisations.

## Introduction

In March 2020, the World Health Organisation (WHO) declared the outbreak of a novel coronavirus (COVID-19) a global pandemic. Following this, countries enforced social distancing and isolation measures as an attempt to mitigate and slow transmission of the virus. The COVID-19 pandemic brought with it a unique set of circumstances whereby many citizens experienced an abrupt loss of income while animal rescue organisations and veterinarians provided restricted services. As a result, companion animals were indirectly affected by the pandemic as people sought company during isolation, leading to an increased interest in pet adoption (Morgan *et al.* 2020; Bennetts *et al.* 2022).

Previous research has shown conflicting results regarding relinquishment and abandonment of animals during the pandemic. In general, rates of companion animal adoptions increased during the pandemic (Morgan *et al.* 2020; Baptista *et al.* 2021; Gomes-Neves *et al.* 2021; Torrico 2021). In some cases, shelters ran campaigns as an attempt to increase pet adoptions (e.g. Royal Society for the Prevention of Cruelty to Animals [RSPCA] Australia’s ‘Clear the Shelter’ campaign; Baptista *et al.* 2021). In contrast, Powell *et al.* (2021) compared statistics from March–June in 2019 and 2020 in 14 Northeastern US shelters and found a decrease in both adoption and relinquishment.

Relinquishment statistics vary between countries, with researchers finding no change in relinquishment in Israel (Morgan *et al.* 2020), an increase in Portugal (Gomes-Neves *et al.* 2021), and a decrease in the USA and Australia (Baptista *et al.* 2021; Powell *et al.* 2021). Morgan *et al.* (2020) reported a correlation between relinquishment and a poorer quality of life index of the owners. An increase in relinquishment could be due to the increase in adoption (Baptista *et al.* 2021) or a potential increase in issues with separation-related behaviours (Holland *et al.* 2021). However, it has also been suggested that pet relinquishment and abandonment increased due to fear of zoonotic transmission (Huang *et al.* 2021; Carroll *et al.* 2022).

Overall, there have been conflicting accounts of increases and decreases in adoption and relinquishment. Perhaps this is due to these studies reporting on a single country’s statistics, and each country and, indeed, region, experienced the pandemic differently. Furthermore, existing research looked at the impact in the months at the beginning of the pandemic prior to most countries entering into additional lockdowns. While these studies provide a good foundation for initial insights into the effect of the pandemic on companion animal rescue organisations, long-term analyses will be necessary to determine any residual effects of the lockdown. Furthermore, there is a lack of research into animal rescue staff and volunteer experiences during COVID-19 (Powell *et al.* 2021), with most studies focusing on the companion animal owners (e.g. Packer *et al.* 2021; Siettou 2021; Carroll *et al.* 2022).

This paper is one in a series of publications that form part of a larger project, ‘CAARP’ (Companion Animal Adoption and Relinquishment during the Pandemic), which seeks to understand adoption and relinquishment of cats and dogs across several countries from the perspective of pet owners, shelter staff, and from shelter records, employing a mixture of qualitative and quantitative approaches to data collection. The term ‘animal rescue organisation’ will be used here to describe those organisations funded by charitable contributions and/or government support to provide care for animals as well as services including animal rescue and adoption.

This paper presents longitudinal research that aimed to explore the impact of the COVID-19 pandemic on companion animal rescue organisations and their staff.

A second aim was to develop a set of recommendations that sought to structure and inspire discussions surrounding how to reduce the risk to companion animal welfare during future similar crises, such as a disease outbreaks, or crises in which there are restrictions on movement and service provision.

## Materials and methods

### Participant recruitment

Data were collected from the 4th of September 2020 to the 23rd of November 2021. The consent form and information sheet were translated into French, Spanish and Italian to increase our reach. However, to avoid translation errors and misinterpretation, English was used for the survey, and participants were informed that the survey itself should be completed in English. Information sheets were translated in the hope of encouraging a greater number of responses from organisations who could then assign an English-speaker to the task of survey completion. A project website was also created for those seeking further information on the aims of the project and the requirements of participation (https://caarpresearchproject.wordpress.com/). It was requested that the survey be completed by a shelter manager or experienced staff member or volunteer. Rescue organisations were recruited through convenience sampling. The study was advertised online on social media platforms (e.g. Twitter), existing contacts were utilised to encourage participation, and calls for participants were made at online meetings hosted by an animal rescue umbrella organisation. In terms of inclusion criteria, both private and public organisations were recruited, which could be registered charities or volunteer groups. There were no restrictions in terms of organisation size. During the data collection process, the terms ‘animal rescue’ and ‘animal shelter’ were used interchangeably, although it is recognised that a distinction is made between these terms in some countries. Municipal pounds and organisations that form part of city and county animal control were not specifically excluded. However, all participating organisations were registered charities. Recruitment activities took place between July 2020 and November 2021. In terms of sample size, a minimum of 15 responses was targeted; Tran *et al.* (2016) resampled data (n = 1,053 patients) from an open-ended survey and found that n = 15 elicited 54% of themes from the original study, and 70% of frequent themes. When data collection concluded, there were 28 complete responses. Tran *et al.* (2016) found that n = 30 samples elicited 69% of themes from their original study, and 86% of frequent themes in the original sample. Therefore, the current sample size is likely to be sufficient to identify most themes.

### The survey

The survey was hosted on Qualtrics. For each shelter or rescue organisation, information on location (country), main funding source, maximum capacity (cats, dogs, other species), and relinquishment fee were gathered. Information on relinquishment fee was collected to assess how this might affect relinquishment. However, there were insufficient data provided on this and relinquishment fee was not considered further. A number of open-ended questions were posed and can be seen in Table 1. The complete survey can be found in the Supplementary material.

**Table 1.** Open-ended questions posed to each animal rescue representative

Open-ended questions
In your opinion, how has the rate of adoptions or interest in adoptions changed since the COVID-19 virus was declared a pandemic (i.e. since 11 March, 2020)? In your opinion, how has the rate of relinquishment and/or abandonment of cats and/or dogs to your shelter changed since the COVID-19 virus was declared a pandemic (i.e. since 11 March, 2020)? In your opinion, what are the main challenges facing animal shelters/rehoming centres as a result of the COVID-19 pandemic? Please provide between one and three challenges. Is there anything the Government, volunteers, or other organisations could do to help at this time and into the future? Do you have any other comments on the impact of the COVID-19 pandemic on your shelter/rehoming organisation and the animals you house?

### Ethical considerations

This study was approved by Queen’s University Belfast Faculty Research Ethics Committee (EPS 20_111).

### Data analysis

Rescue organisation demographic information was analysed using descriptive statistics in SPSS. Five key open-ended questions were posed (Table 1, see Supplementary material for the complete questionnaire). The questions were devised after a review of the white and grey literature surrounding the effects of COVID-19 on companion animal adoptions and relinquishment. At the time of data collection, the effects of COVID-19 in this regard had been reported anecdotally, or at the individual organisation level. Therefore, questions one and two were deemed important to include. Given the unprecedented situation, question three aimed to gather experience of the specific challenges faced by rescue organisations during the pandemic and associated lockdowns. Question four sought to identify solutions for future use, while question five was added to ensure that no important points were missed by asking participants to add anything else they deemed important. Quantitative data were also collected from rescue organisations and are presented elsewhere (Carroll *et al.* in prep). The questionnaire’s open-ended questions were organised and analysed in NVivo 14 following a thematic approach outlined by Braun and Clarke (2006). More specifically, descriptive thematic analysis was used to identify recurrent themes within the data. Analysis was carried out across questions, rather than for each question individually, in order to identify patterns running throughout the dataset as a whole. We used an inductive approach where codes and themes were developed from the actual data, rather than assigning *a priori* codes. In practice, this involved an iterative process where, as new codes were generated, they were checked for redundancy against existing codes, and then grouped into overarching themes. Our themes were generated hierarchically through three levels of analysis (Table 2). At the lowest level are the codes we found across the data set. Then, at the second level, subthemes were identified by grouping codes which shared an underlying meaning. At the highest level, we grouped the sub-themes into overarching themes which provided a global, more abstract view of the data.

**Table 2.** Table of codes and overarching sub-themes and themes. The frequency of each code is provided to show to how many shelter responses (out of 28) had this code

Theme	Sub-theme	Codes	Overall	By organisation							
					**1**	**2**	**3**	**4**	**5**	**6**	**7**
Impact on animals	Change in owner circumstances/ attitudes	Best time to adopt	6	6	6	0	0	0	0	0	0
		Lockdown changed owner behaviour/attitudes	1	1	1	0	0	0	0	0	0
		People changing their minds	5	5	5	0	0	0	0	0	0
		Change of circumstances	3	3	1	1	1	0	0	0	0
		Relinquished because of fears of carrying virus	1	1	1	0	0	0	0	0	0
	Effect on adoption	Decrease in adoptions	6	6	4	0	0	1	0	1	0
		Increased demand for pets	14	14	12	0	1	0	1	0	0
		Increase in adoptions	22	20	17	1	1	1	1	1	1
		No change in adoptions	4	4	2	0	0	0	2	0	0
	Effect on relinquishment	Decrease in relinquishment	15	15	15	0	0	0	0	0	0
		Increase in relinquishment	16	16	11	1	2	0	1	1	0
		No change in relinquishment	6	6	3	0	0	1	1	0	1
	Benefits to animals	Less stress from fewer visitors	4	4	3	0	0	0	1	0	0
		Staff can spend more time with animals	3	3	3	0	0	0	0	0	0
		Unpopular breeds more likely to get adopted	1	1	1	0	0	0	0	0	0
	Risks to animals	Increased cost and risk of buying vs adopting	2	2	2	0	0	0	0	0	0
		Lockdown influences animal behaviour	4	4	3	0	0	0	0	0	0
		Longer holding times	2	2	2	0	0	0	0	0	0
		Vet clinics not open	13	12	10	0	1	0	0	0	1
Impact on identity	Funding	Help with funding	12	12	7	1	0	1	2	0	1
		Lack of funding	24	24	18	2	0	0	3	1	0
	Legality	Lockdown highlighted need for rule changes	5	5	1	0	1	0	1	2	0
		Maintaining safety of staff and customers	10	10	8	0	0	0	1	0	1
		Unclear and rapidly changing and COVID-19 guidelines	11	11	10	0	0	1	0	0	0
	More publicity and appreciation	Felt overlooked as an essential service	5	2	2	0	0	0	0	0	0
		Wished for more acknowledgement	3	3	3	0	0	0	0	0	0
Impact on organisational processes	Improving the success rate of adoptions	Educating owners	10	10	7	0	0	1	1	0	1
		Pandemic allowed procedural changes to be made	2	2	1	0	0	0	0	0	1
		More time spent picking the right owner	8	8	6	0	0	1	1	0	0
	Mental health and exhaustion	Decreased mental health of staff	5	5	5	0	0	0	0	0	0
		Fewer volunteers	9	9	7	1	0	0	0	1	0
		Overcame the challenges as a team	4	4	5	0	0	0	0	0	0
	Online and illegal sourcing and sales	Raise awareness of puppy farmers/unreputable breeders	3	3	3	0	0	0	0	0	0
		Tighter controls on scams and online sales	3	2	2	0	0	0	0	0	0
		Tighter controls on smuggled pets	1	1	1	0	0	0	0	0	0
	Working with other organisations	Sharing resources	3	2	2	0	0	0	0	0	0
		Promoting co-operation across sector	2	2	2	0	0	0	0	0	0

1 = United Kingdom, 2 = Republic of Ireland, 3 = France, 4 = Spain, 5 = Germany, 7 = Australia, 8 = USA

## Results

### Descriptive statistics

After removal of incomplete responses, data from n = 28 rescue organisation branches were available for analysis. Nine single-site organisations provided data, and two larger organisations provided data for five branches each. A total of 67.9% (n = 19) of participants were based at an animal shelter or rescue organisation in the UK, 14.3% were based in Germany (n = 4), and five were from each of the remaining countries: the Republic of Ireland, France, Spain, USA and Australia. The main funding source for the organisations were donations/legacies (89.3% of organisations), government support (7.1% of organisations), and adoption fees (one organisation). In total, of the 28 rescue organisations, 22 housed cats (two exclusively housed cats), 24 housed dogs (ten exclusively housed dogs) and 18 housed other species in addition to cats and dogs. The mean (± SD) shelter capacity for cats was 82.4 (± 108.47); (range: 8–539), for dogs 85.04 (± 118.74); (3–587) and for animals belonging to other species 53.9 (± 99.25); (10–430). In relation to incoming animals, 59.1% of organisations made a distinction between relinquishments and abandonments, while 36.4% did not. For one organisation, this question was not applicable. In total, 40.9% of organisations charged a relinquishment fee.

### Qualitative results

**Figure 1.**
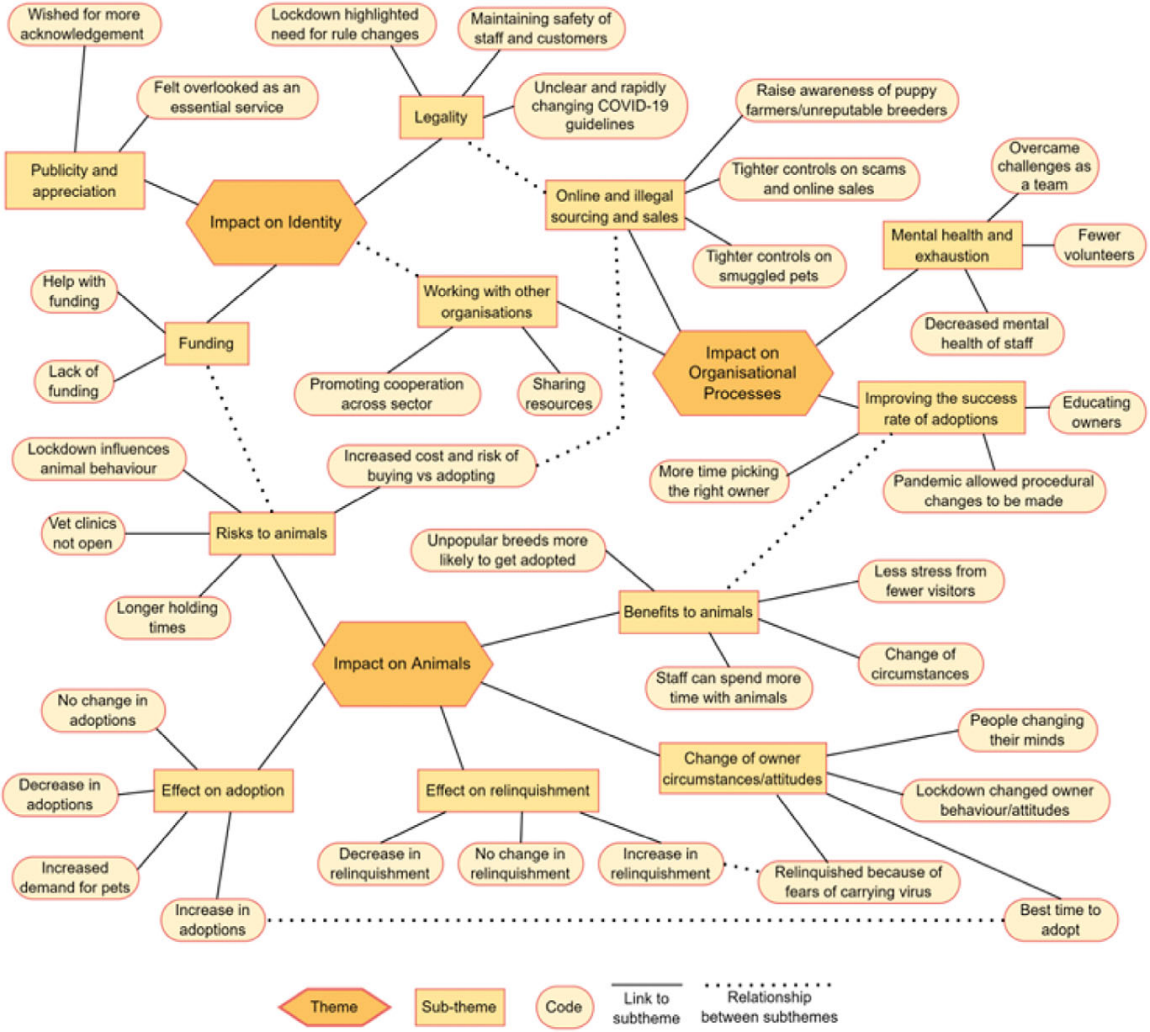
Thematic map showing the multi-dimensional relationships between the various themes, sub-themes and codes.

Overall, we identified three themes related to accounts of how the COVID-19 pandemic affected shelters: (1) impact on animals; (2) impact on identity; and (3) impact on organisational processes. In this section, we describe and illustrate these themes with narrative examples. To show how the overall themes were created, Table 2 shows the breakdown of themes into sub-themes alongside examples of representative quotes coded into that theme. Figure 1 shows the thematic map which displays the relationships between the identified themes, sub-themes and codes.

### Impact on animals

Reports from animal rescue staff and volunteers suggest that animals were affected both positively and negatively by the pandemic, through consequences from lockdown restrictions and subsequent changes in adoption and relinquishment rates. There were perceived changes in adoption and relinquishment in nearly all shelters, with many reporting increases in adoption and overall demand for pets. For example, one participant noted, “*before the pandemic we had 2–4 requests for dogs a week, through the pandemic we had up to 100 requests a week*”. In general, the demand for pets remained consistent throughout the pandemic. Several participants thought this was because people thought it was the best time to adopt as owners were at home with more time to spend on a pet. However, this made some wary as they were aware of people who “*just want a pet for lockdown or to keep children occupied*”, with another saying that “*many hadn’t even considered getting a pet before the lockdown period and when information was sought about what their position was after lockdown no one had much of an answer to make us comfortable rehoming to them*”. Their concerns were realised as relinquishment rates were reported to escalate upon the ending of each lockdown. Many rescue organisations reported this pattern in relinquishment rates, with rates decreasing at the start of, and during, lockdowns and then dramatically increasing as lockdown ended. For example, the UK entered its first lockdown between March 2020 and June 2020 and one UK rescue centre said that “*the rate of relinquishment dramatically reduced during the first lockdown period* [until July 2020] *to such an extent that we no longer had a waiting list and empty kennels*”. During the summer months, there were only limited restrictions and another rescue centre reported “*an increase in request to relinquish between July and September*”. November 2020 marked the second UK lockdown and one participant stated that as that lockdown came to an end, “*in the last couple of weeks there has been a big increase* [200%]”. Similarly, large increases in relinquishment were reported shortly after the final lockdown ended: “*since June 2021 there has been an increase in people needing to give up their dog or cat, for a number of reasons*”.

Many pets were reported to have been relinquished as people returned to normal life, leading rescue organisations to say that they “*saw some of the worst cases of abuse and neglect*” of dogs and that “*behavioural issues have increased in both number and severity*”.

One rescue organisation mentioned the increase in behavioural issues may be due to “*owners being around the animals much more during lockdown*”. Another effect of lockdown was the closure of veterinary practices for ‘non-emergency services’, such as neutering and spaying. Many participants reported a significant increase in pets being relinquished who had not been spayed or neutered and “*pets with more complex behaviour and medical needs, where owner* [sic] *have struggled to access veterinary services*”. For cats, in particular, rescue organisations found more cats being abandoned as opposed to relinquished, stating that owners became overrun as “*many cats were unable to be sterilised as vet clinics closed their doors, so many more cats were born, those cats have had litters”.* One participant also thought that this pattern may be due to “*various news of cats being carriers of the virus. We have seen a rise each time their* [sic] *is a spike in cats being a cause or risk factor with regards to COVID – and our belief is that many are being abandoned due to fears of the owners*”. The travel restrictions imposed during lockdown resulted in rescue organisations stating that animals were held for longer than needed. For example, “*some were required to stay in our care until some of the restrictions were lifted and they could have a visit that was serval* [sic] *months* [long]”.

However, the pandemic led to certain benefits for animals. Due to the lockdown, members of the public were unable to visit shelters, meaning “*staff have had more time to spend working with the dogs*”. Many participants also said that animal welfare was improved: “*having less visitors in the rehoming centre has greatly improved the dogs’ welfare. Not being on show to the public has taken that stress away*”. One rescue found that unpopular breeds were more likely to be adopted as “*the increased demand from the public for dogs has helped us rehome some of our long-term dogs to good homes. Breeds have been considered that wouldn’t have been in previous years*”.

It is worth noting that the above reflects the experiences of those in the rescue centre/organisation and may or may not be an accurate reflection of the quantitative intake and adoption figures.

### Impact on identity

The pandemic impacted how rescue organisations perceived their image and governmental standing. Rescue organisations felt that rules they imposed during the pandemic were too vague and needed to be “*more specific on where ‘animal welfare’ organisations fit within guidelines; especially travel and necessity*”. This caused confusion within the organisations as they are “*trying to stay within restrictions but* [we’re] *unsure which areas we fall under as each person has a varying opinion*”. Rules also differed across borders which created further confusion. For example, one participant said: “*we are based on the England/Scotland border and this has caused various issues. We are finding those contacting us do not understand or choose to ignore the Government guidelines for themselves*”. Another issue raised by several rescue organisations was the difficulty in implementing social distancing and maintaining the safety of their workers. For some, this meant they were “*working with a much smaller team to maintain social distancing*”. Indeed, where teams were small to begin with, there were concerns around “*keeping staff safe so we can keep the sanctuary operating*”. Some rescue organisations highlighted the need for flexibility in the lockdown rules, for instance “*flexibility re: online adoptions and contactless animal drop-offs to their new homes*”. Rescue organisations also gave recommendations for additional rules, such as to “*oblige vet clinics to stay open and offer low price neutering*”. Other suggestions included tighter controls for microchipping and allowing more people to temporarily foster animals if they are not able to adopt long term.

The greatest challenge, which was raised by virtually every respondent, was the lack of funding. This arose from restrictions on normal fundraising practices as well as a lack of monetary support (e.g. grants) from the Government. This not only negative affects staff, but also has an indirect effect on the animals themselves. For example, one participant stated that “*most charities are experiencing deficits this year and looking at job cuts, reducing services and this really will not help the cats out there who need us*”. Many also mentioned a lack of funds from the owners’ perspectives as “*loss of income has affected ability for owners to pay for some vet costs*”, causing further potential risk to animals. In the future, rescue organisations hope for help from the Government in the form of grants and funding to offset the effect from decreased fundraising. For example, as facilities were closed to the public, “*many of us relay* [sic] *on people visiting the organisation and seeing the animals and making a donation – with restrictions we cannot do that*”. Managers further expressed that they felt overlooked as other animal care services were receiving governmental support: “*Grants for animal rescues, like zoos obtained, Why have we been left out?*”.

This feeling of being left out was also prevalent throughout many of the responses in regard to Government policies and lockdown rules:

*“We need to know where we stand in the eyes of the government. There needs to be thought given to charitable organisations working with animals; we are an essential service [no matter what some people believe] – if we do not work then animals suffer and people suffer. We provide a lifesaving service but so often we are overlooked. We have taken in animals whose owners have passed away, or have been hospitalised at a time when we were supposed to be in lockdown. We have been pulled over by the police and questioned when we have been transporting animals or going to the vets. We don’t know where we stand in these situations”.*

Several participants stressed how challenging the staff had found working during the pandemic. They praised the dedication of their staff and their commitment to caring for the animals as *“the animals always come first and truthfully not one has suffered a loss of care in this time, thanks to the dedication of the people providing care”.* Rescue centres struggled as their caregiving services were largely impacted due to lockdown restrictions:

*“The pandemic has caused exhaustion, depression, desperation, – all through trying to work under conditions that are unknown and feel like at every turn someone is trying to stop you or impede your work. Many animal rescues* [sic] *stopped working during lockdown – we restricted our activities but if we had stopped working then there would be over 240 animals who would have not be* [sic] *helped”.*

However, these efforts often went unnoticed, and one participant said that *“greater new* [sic] *stories on how charities like us were working through the pandemic would have been benefical* [sic]*”.*

### Impact on organisational processes

There were many guidelines and rules imposed during the pandemic and these significantly impacted rescue services and processes. However, this was not always negative. Some shelters found the pandemic to also have a positive effect on their processes. For example, *“in many ways, the pandemic was very good for my organisation. It allowed us to stop and make decisions based on real reasons rather than history or ‘because we’ve always done it that way’".* A few rescue organisations recalled positives as they reflected on how they overcame the challenges of the pandemic as a team. For instance, one said they were *“pleasantly surprised that our charity has been able to adapt quickly and easily using digital alternatives to continue supporting pets and owners”.*

In general, however, rescue organisations found it difficult to operate under the various restrictions and rules. Regarding adoption applications, rescue organisations experienced an *“increased workload with high number of applications for each dog and managing customer expectations”.* The increase in requests also brought about an increase in unsuitable applications, such as *“time-wasters or inappropriate rehomers”* or *“adoptants who just look for an animal to avoid restrictions”* which further added to the workload. To keep ensuring that prospective homes were suitable, one manager explained how they had tried to adapt but instead put new applications on hold:

*“we started doing virtual home checks to new applicants but later stopped this as we didn’t feel it was as affective as a proper home visit. We stopped doing these and kept applications on hold until lockdown ended so that we could carry out home visits in peoples* [sic] *gardens. We used applicants who were home-checked prior to lockdown hitting as a way of continuing to rehome”.*

To combat this, many shelters spoke about improving owner education and awareness of certain issues, such as leaving your pet alone after lockdown and whether it is the right time to get a pet. They found that during lockdown, *“the dogs we see that are coming into the shelter are not spayed or neutered, not socialised, have been bought from various places which have given the new adopter no advice or guidance when buying the dog”.* One participant advised to *“increase public education resources/knowledge on health and welfare standards of dogs over the increasing consumer-based behaviour of obtaining a pet ‘on demand’”.* Several rescue organisations noted the importance of *“helping adopters understand* [the] *need to get dogs used to being by themselves”* and to *“continue to provide advice to owners to help prepare their pets for when they are left for longer hours when they return to the office”.*

Some shelters found that they had *“rehoming competition from pet sales websites and breeders”.* In response, they highlighted the need for tighter controls and awareness of illegal sales and scams. For example, one participant explained, *“with the high demand for dogs there has been a rise in smuggled pups into the country. Tightening up on this and increasing the penalties would help to put a* [sic] *end to this practice”.* Other shelters argued for the increased *“legislation around breeding of animals – puppy farming etc”* and *“tighter control on online sales of pets cracking down on the scams”.* Shelters noted a significant decrease in staff morale and mental health during the pandemic. This was due to a variety of factors, such as fewer volunteers and staff, being overlooked by Government, and a lack of understanding from applicants and the public. For example, one manager recalled that:

*“our supporters have been great but we have faced backlash from people who we refused to rehome to [for reasons that we stand by] or from people who rang but we didn’t answer the phone to – even after repeated explanations that our office is unmanned. These simple comments hit hard on already frayed and tattered minds, and we worry for the mental health of some of our volunteers and staff at this time”.*

The constant restrictions and length of lockdowns have also taken their toll on the mental health of staff:

*“Initially staff have been very positive about coming to work and focusing more time with the dogs, however during the most recent lockdown* [November]*, staff morale has dipped due to poorer weather and a feeling that the pandemic restrictions will ‘never end’. Staff have worked really hard throughout this pandemic and some are feeling very fatigued, and frustrated when they know friends and family that have been on furlough through the period and not had to work under such stressful conditions”.*

Staff morale also suffered due to a decrease in staff and volunteers. Social distancing restrictions and other safety restrictions led to fewer volunteers being able to work. Several rescue organisations said that *“we are all exhausted due to the sheer amount of work the reduced staff and volunteers have to do”.* The decrease in volunteers had a significant impact on some rescue organisations as *“we have a small team, a team who have worked to the point of exhaustion and beyond and it feels sometimes that there is no end. It is very wearing on our mental health”.*

Participants expressed their desire to work with other organisations to overcome the challenges of the pandemic and in general, such as for challenging cases. It *“would be great for charities to communicate between each other. If they have a high welfare case needing help with, they work together as a sector to get them sorted”.* Other rescue centres felt there was the potential for co-operation by *“sharing volunteers, especially for roles we find hard to fill [drivers etc]”* and to *“help movement of pets and help with space and capacity”.*

## Discussion

Through investigating the lived experiences of shelter managers, we have identified the main challenges experienced by shelters during the COVID-19 pandemic. Despite losing their primary sources of funding and working with reduced staff, animal rescue organisations worked to continue to feed, rescue, and care for animals throughout the pandemic. Prior work has also found many animal rescue organisations struggled with funding and revenue loss during the pandemic due to the closure of charity shops, and cessation of fundraising events (Baptista *et al.* 2021; Torrico 2021). Funding may have decreased. However, staff workloads escalated, with the majority of shelters reporting a dramatic increase in adoption requests. Ho *et al.* (2021) reported that Google searches for pet adoption increased by up to 250% in 2020 compared with 2019, peaking in April and May 2020. In the current study, shelter staff speculated this was due to people perceiving it to be an optimal time to adopt as they had more time to spend at home. This supports findings from Morgan *et al.* (2020) who reported that, in Israel, people were motivated to adopt during the pandemic as they had extra available time or to reduce stress or loneliness. This is echoed by Bennetts *et al.* (2022) who found that one-fifth of Australian families had acquired a new pet during lockdown as a result of spending more time at home.

There have been conflicting reports of relinquishment and adoption over the pandemic. Studies have found a general increase in relinquishment (Gomes-Neves *et al.* 2021), while some have found no change overall (Morgan *et al.* 2020). Respondent reports on adoption and relinquishment during the pandemic suggest that there may be a trend in increased relinquishment rates. In our longitudinal study, we highlighted a pattern of relinquishment over the pandemic where relinquishment rates were perceived to have increased as lockdown restrictions eased. While Powell (2021) found that fewer dogs and cats were admitted to and adopted from animal rescue centres, their study was conducted at the beginning of the pandemic from March–June 2020. This supports the idea that relinquishment rates decreased as restrictions increased. We also found that, with a perceived increase in relinquishment, an increase in behavioural and health problems among the pets relinquished to shelters was reported. For example, separation-related behaviour is one of the most common reasons given for relinquishment (Segurson *et al.* 2005). With owners spending more time at home with their pets, Holland *et al.* (2021) warned of a potential increase in relinquishment after lockdown due to the issue of separation-related behaviours. Considering the above, it is worth noting that qualitative experiences may differ from quantitative evidence. Qualitative research involves collecting information on people’s experiences, while quantitative research depends on numeric data (Ahmad *et al.* 2019). Often, qualitative and quantitative results are incongruous (Wagner *et al.* 2012). Therefore, the results presented here should be interpreted carefully. Intake and adoption figures from animal rescue centres are explored in a separate publication (Carroll *et al.* in prep) and will provide an insight into how human perceptions align with hard numbers in relation to animal intake and adoption post-pandemic. As well as negatively impacting the animals, the pandemic also increased the risk to shelter staff’s mental health. In addition to revenue losses, our results revealed that for the majority of lockdown, staff were also working under increased workloads due to an influx of adoption requests from the public, and increased relinquishment requests as lockdowns ended. Similar to the general populace, the mental health of staff and general morale were found to be heavily affected by the pandemic. This supports the work of Dalton *et al.* (2022) who found that 89% of workers in the animal care and veterinary profession were concerned about mental health implications for staff as a consequence of the pandemic. We also found that shelter staff were under further stress from ambiguous lockdown rules. Staff reported a disconnect between the need to continue to perform essential services whilst also having to adhere to restrictions imposed by the Government, such as travelling to collect animals of owners who have passed away or been hospitalised with COVID-19. Shelters felt that they inhabited a ‘grey area’ when seeking to operate under the restrictions imposed during lockdown. This is in accordance with the findings of Gomes-Neves *et al.* (2021) who revealed that Portuguese municipal shelters were most affected by the lack of instructions. Along with the lack of help with funding, the struggle to follow local restrictions perpetuated the feeling of being ignored and underappreciated by the authorities and the Government.

### Recommendations

Rescue organisations reported a wide range of factors that exacerbated the stress of operating during the pandemic. Drawing from the rescue organisations’ responses, we have created a list of recommendations regarding possible measures to be put in place that would reduce the risk to animals and shelter staff during a crisis such as the COVID-19 pandemic (for an overview of the key recommendations, see Table 3).

**Table 3.** Recommendations to reduce the risk to animal welfare, and rescue staff well-being, during a crisis

Key recommendations	Sub-recommendations	Relevent to
Promote cooperation and modifications within the sector	Resource sharing	Rescue organisations
	Working together on challenging cases	
	Implement an emergency foster care system for people who are not eligible to adopt long term but still want to help	
	Develop adoption protocol for risk-assessing owners remotely during a crisis	
Increased education of the general public	Increase awareness of the work animal rescue organisations do	Rescue organisations Researchers Governments and policy-makers
	Educating owners on bad breeders and online scams	
	Increase owner education of adopting a pet and behavioural issues – e.g. socialisation during lockdown and separation anxiety after lockdown	
Clarifying the identity of animal rescue organisations within the Government	Acknowledge the life-saving work of shelters	Governments and policy-makers
	Clarify where shelters stand in relation to other animal services	
	Ensure there are clear rules for shelters workers	
	Ensure essential veterinary services are available to shelters during a crisis – i.e. spaying and neutering	

### Promoting co-operation and change within the sector

Rather than creating new problems, crises and emergency situations often simply expose underlying systemic issues (Heath *et al.* 2015; Onukem 2016). The COVID-19 pandemic brought a number of shortcomings, in terms of organisational processes, to the surface. In the current study, the effects of the COVID-19 pandemic on relinquishment varied across organisations; 15 reported a decrease in relinquishment, 16 reported an increase, and six reported no change in relinquishment, compared to pre-COVID. This suggests that rescue organisations had different experiences of the pandemic, with some struggling in response to the pandemic, when others did not. This variation is also found in other recent studies on the subject (e.g. Morgan *et al.* 2020; Baptista *et al.* 2021; Gomes-Neves *et al.* 2021; Powell *et al.* 2021).

Animal rescue organisations vary in their size, scope, income, and in the number of staff compared to volunteers (Vinic *et al.* 2020). Animal rescue organisations also differ in the welfare problems that they encounter. For example, rural shelters may be more inclined to encounter reduced spaying and neutering within the community, reduced availability of veterinary services, and generally high levels of pet-ownership (Ly *et al.* 2021a; Horecka & Neal 2022). These differences may impact organisations’ ability to cope in unprecedented situations. Considering these varying pressures, animal rescue and shelter organisations would benefit from pooling resources in times of crisis. Indeed, it has been argued that there is a shared responsibility to act in emergency or unprecedented situations (Travers 2022). While animal rescue organisations can benefit from working together in challenging times, this is something that organisations would benefit from more broadly.

Attempts have been made to co-operate across organisations. For example, the British Columbia Society for the Prevention of Cruelty to Animals (BC SPCA) transfer animals internally across 34 branches (Gordon *et al.* 2020; Ly *et al.* 2021b). Transfer of animals between branches is done to increase the chances of animals being adopted by moving them from busier branches to those with greater capacity to take incoming animals (Ly *et al.* 2021b). Such a system could work *between* organisations. However, an agreed procedure and set of rules for inter-organisation transfers would be required. There have also been attempts to share data between organisations. However, transfer of information on animals can be lacking and, to date, there has been relatively poor uptake of between-organisation initiatives (Vinic *et al.* 2020; Horecka & Neal 2022). This may suggest a somewhat reactive response to unexpected obstacles. The COVID-19 pandemic forced many shelters to re-evaluate their processes and the nature of their relationships with other organisations. More research is needed to assess ways to share resources and best practice.

### Increased education of the general public

Despite the wide availability of information today, pet owners often make poor choices, from carrying out insufficient research prior to acquiring a pet, to failure to adequately train their companion animals. In the current study, animal rescue staff and volunteers highlighted the need for increased education of the public on various important topics. Indeed, many animal rescue organisations, veterinarians and researchers have called for increased education of companion animal owners (Philpotts *et al.* 2019; Murphy *et al.* 2022). Furthermore, the increase in first time pet-ownership around the time of the COVID-19 pandemic makes education of pet owners of particular importance moving forward (Murphy *et al.* 2022). For example, Carroll *et al.* (2022) found that 63.2% of people that had relinquished a cat or dog were first-time pet owners, suggesting that lack of experience and knowledge may contribute to undesirable outcomes for companion animals. However, owners may possess knowledge of best practice, yet they may not exhibit corresponding behaviour based on this knowledge.

It is increasingly recognised within the animal welfare science community that education alone is insufficient in changing the attitudes and behaviour of cat and dog owners (Glanville *et al.* 2020). Indeed, education is sometimes viewed as a ‘fix-all’ solution to addressing animal welfare problems (Philpotts *et al.* 2019). Rather than solely focusing on education of the public, further research is needed to explore various facets of pet owner behaviour that affect decision-making (Kuhl *et al.* 2021). In addition to education, avenues including training and incentivisation should be explored (Michie *et al.* 2011; Carroll & Groarke 2019). For instance, while we should aim to increase pet-owner knowledge of how to identify an online scam, other influences on behaviour, including social influences and the opportunity to acquire a cat or dog via a reliable source should also be considered. In the current study, pet-owner behaviour has been identified as key to improving the welfare of cats and dogs, not only in the shelter environment, but in the home. Identifying effective ways to target cat and dog owners will require collaboration between researchers, animal rescue organisations and policy-makers. In particular, approaches are needed to increase the ability of pet owners to identify and avoid bad breeders and online scams, prepare prospective owners for the realities of owning a pet, and make behaviour training accessible to dog owners. While not a simple task, a holistic approach is necessary to address topical welfare issues affecting companion animal species.

### Clarifying the identity of animal rescue organisations within the Government

While consensus could be reached on the essential nature of supermarkets, healthcare provision and rubbish collection during the COVID-19 pandemic, the importance of other services is more subjective, and varied according to local needs (Storr *et al.* 2021). In the Republic of Ireland, veterinary, animal welfare and related services were deemed essential, including at the highest level of restriction, when rates of COVID-19 infection were very high (Gov.ie 2020). In the US, animal shelter and rescue organisations were also declared essential services. However, services were restricted to those deemed to be most crucial, and regulations varied across states (Szydlowski & Gragg 2020). Similarly, in the UK, pet animal and veterinary businesses were deemed essential from the 23rd of March 2020 (RSPCA 2020). Despite this, the Association of Dog and Cat Housing (ADCH 2020) found that COVID-19 affected the ability of 97% of UK and Irish rescue organisations to operate effectively and over half of the surveyed organisations reported reduced access to essential veterinary care. In the current study, several participants referred to unclear and changing COVID-19 guidelines, the feeling of being overlooked as an essential service, and the inability to access veterinary services. Greater clarity and guidance are required, as well as financial support, and a recognition that those working in the animal charities’ sector have an important role to play in society.

### Limitations and future directions

In the current study, the majority of participating organisations were based in the UK. This limits the generalisability of the data. Efforts were made to recruit internationally, for example, by translating participant information sheets into three additional languages, and contacting organisations from several countries. Translating the survey into a number of languages may have increased our response rate and should be considered in the future. While a larger sample size was desirable for the current study, and some themes may have been missed, similar themes emerged across the organisations regardless of geographical location. Future research projects in this area should involve collaboration with a team of international researchers, as local knowledge and connections will aid recruitment.

Further research is needed to review successes and failures in changes to organisational processes during the COVID-19 pandemic. To avoid reactive response to emergencies in the future, it would be beneficial to establish protocols for animal rescue organisations when working during an emergency or crisis, from emergency adoption protocols to fostering systems, and cross-organisation co-operation. To reduce the workload of animal rescue organisations in the long term, pet-owners’ behaviours that negatively impact companion animal welfare should be targeted for intervention. The findings of this research should also be considered by Government bodies when developing plans for future emergencies.

### Animal welfare implications

The current study identified key challenges faced by cat and dog shelters during the COVID-19 pandemic, including the impact on animals, the impact on identity of rescue organisations and their staff and volunteers, and the impact on organisational processes. Negative effects of the COVID-19 pandemic and associated lockdowns were identified, including animal behaviour problems and decreased mental health of staff. Importantly, however, not all challenges and experiences had a negative outcome. For example, rescue staff and volunteers reported less stress in animals as a result of having fewer visitors, and rescue organisations could afford to spend time picking the right owner. Based on the experiences of shelter staff and volunteers, recommendations were made for future crises, and indeed standard practice moving forward. These focus on the need to promote co-operation and change within the sector, increase education of the general public, and the need for clarification regarding the identity of animal shelter animal rescue organisations within the Government. These recommendations, including the modification of practices and relationships within the sector, and addressing pet owner behaviour, provide a starting point for improving the welfare of cats and dogs housed in shelters, both during a crisis and more generally.

## Supporting information

Carroll et al. supplementary material Carroll et al. supplementary material

## Supplementary material

The supplementary material for this article can be found at http://doi.org/10.1017/awf.2024.15.
