# Supplementary material for: Companion animal adoption and relinquishment during the COVID-19 pandemic: The experiences of animal rescue staff and volunteers
Source: Anim Welf. 2024 Mar 4;33:e12. doi: 10.1017/awf.2024.15 (PMC10951665; doi:10.1017/awf.2024.15)
Supplement: Carroll et al. supplementary material [file S0962728624000150sup001.pdf]

Companion animal adoption and relinquishment during the COVID-19 pandemic: The experiences of animal rescue staff and volunteers

GA Carroll, C Reeve, A Torjussen

Questionnaire

1. What is the name of the shelter for which you are completing this survey?

---

2. In which country does your shelter/rehoming centre operate?

○ Canada (1)

○ USA (2)

○ United Kingdom (3)

○ Ireland (4)

○ Australia (5)

○ New Zealand (6)

○ Italy (7)

○ Spain (8)

○ France (9)

○ Other: (10) ____

3. Where does the majority of your funding come from? Select the option that is most applicable.

○ Government support (1)

○ Donations and/or legacies (2)

○ Shops associated with the shelter (3)

○ Other (please specify): (4)
____

---

4. What is your maximum capacity (i.e. the maximum number of animals that you can house at any one time)? If this does not apply to your organisation, you can leave this question blank.

Maximum number of cats: (1)

Maximum number of dogs: (2)

Maximum number of other species: (3)

---

5. In your opinion, how has the rate of adoptions or interest in adoptions changed since the COVID-19 virus was declared a pandemic (i.e. since 11 March, 2020)?

---

6. Does your organisation differentiate between animals that are relinquished (brought to your facility by the owner or guardian and an agreement is made to leave the animal with the shelter) versus animals that are abandoned (animals that are left at the shelter without any agreement with the owner/guardian)?

○ No (1)

○ Yes (2)

○ Does not apply to my organisation (3)

---

7. Does your organisation charge a fee for relinquishing a cat or dog? If yes, how much?

○ No (1)

○ Yes. Our relinquishment fee is: (2)

---

8. In your opinion, how has the rate of relinquishment and/or abandonment of cats and/or dogs to your shelter changed since the COVID-19 virus was declared a pandemic (i.e. since 11 March, 2020)?

   (If you organisation differentiates between animals that are relinquished versus abandoned, please comment on both)

---

9. In your opinion, what are the main challenges facing animal shelters/rehoming centres as a result of the COVID-19 pandemic? Please provide between one and three challenges.

○ #1 (1) ____

○ #2 (2) ____

○ #3 (3) ____

---

10. Is there anything the government, volunteers, or other organisations could do to help at this time and into the future?

---

11. Do you have any other comments on the impact of the COVID-19 pandemic on your shelter/rehoming organisation and the animals you house?
